# Crystal structure of nitridobis(tri­methyl­silanolato)[1,1,1-trimethyl-*N*-(tri­methyl­sil­yl)silanaminato]molybdenum(VI)

**DOI:** 10.1107/S2056989015021192

**Published:** 2015-11-14

**Authors:** Caiwei Geng, Xiang Hao, Peng Jiao

**Affiliations:** a College of Chemistry, Beijing Normal University, Beijing 100875, People’s Republic of China; b Institute of Chemistry, Chinese Academy of Sciences, Beijing 100190, People’s Republic of China

**Keywords:** crystal structure, molybdenum complex, nitride, tri­methyl­silyl oxide, hexa­methyl­disilazide

## Abstract

In the title compound, [Mo(C_6_H_18_NSi_2_)(C_3_H_9_OSi)_2_N], the Mo^VI^ cation is located on a mirror plane and is coordinated by a nitride anion, a 1,1,1-trimethyl-*N*-(tri­methyl­sil­yl)silanaminate anion and two tri­methyl­silanolate anions in a distorted tetra­hedral geometry; the N atom and two Si atoms of the 1,1,1-trimethyl-*N*-(tri­methyl­sil­yl)silanaminato anionic ligand are also located on the mirror plane. The Mo N bond length of 1.633 (6) Å is much shorter than the Mo—N single-bond length of 1.934 (7) Å. No hydrogen bonding is observed in the crystal structure.

## Abstract 2

In the title compound, the Mo^VI^ cation is located on a mirror plane and has a distorted tetra­hedral coordination geometry. The Mo N bond length is 1.633 (6) Å.

## Chemical context   

The title compound, nitridobis(tri­methyl­silanolato)[1,1,1-trimethyl-*N*-(tri­methyl­sil­yl)σilanaminato]molybdenum, is a precursor for the preparation of nitridotris(tri­phenyl­silanolato)molybdenum, which can generate alkyl­idyne­tris(tri­phenyl­silanolato)molybdenum, a superbly active catalyst for alkyne metathesis reactions (Bindl *et al.*, 2009 ▸; Heppekausen *et al.*, 2010 ▸). The structure of the title compound has been characterized by IR, ^1^H and ^13^C NMR and low resolution MS spectroscopy (Chiu *et al.*, 1998 ▸; Bindl *et al.*, 2009 ▸; Heppekausen *et al.*, 2010 ▸). However, to our knowledge no crystal data have been reported because the title compound is an oil at room temperature, and is highly sensitive to air and moisture.
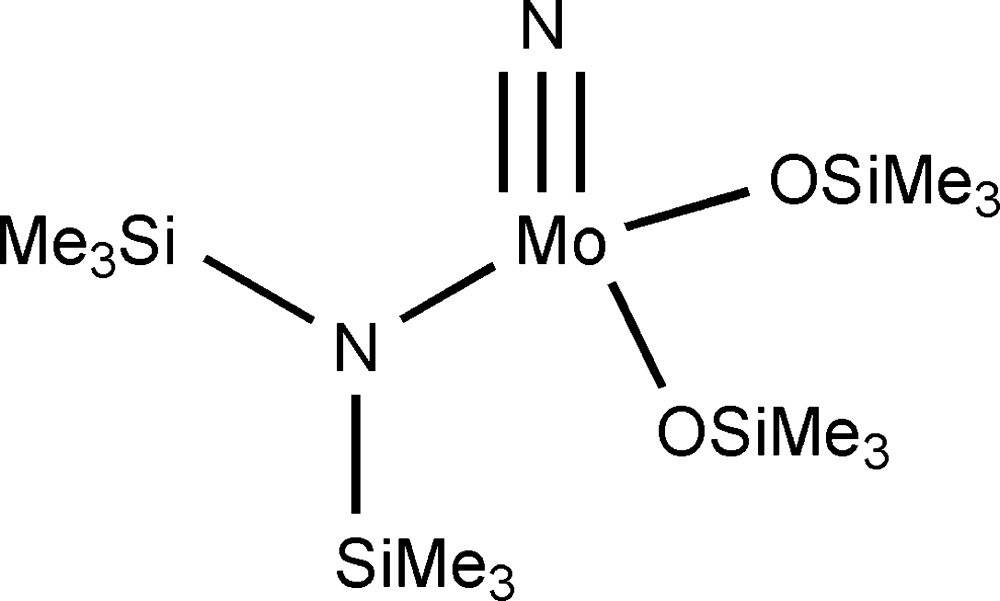


## Structural commentary   

**Figure 1:**
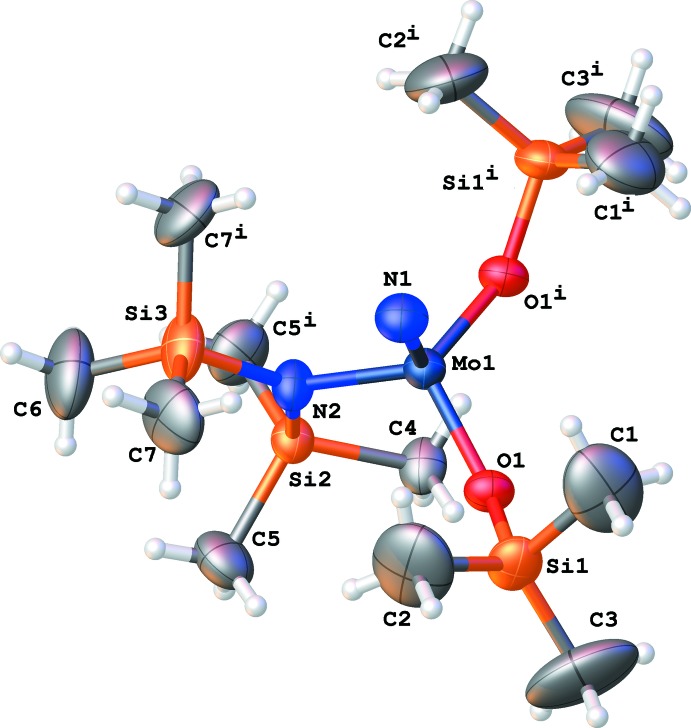
The crystal structure of the title compound. Displacement ellipsoids are drawn at the 30% probability level. [Symmetry code: (i) *x*, 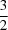 − *y*, *z*.]

In the crystal, the title complex (Fig. 1 ▸) resides on a crystallographic mirror plane, therefore, the asymmetric unit consists of half of the complex. Atoms Mo1, N1, N2, Si2, Si3, C4, and C6 lie on the mirror plane. The compound is a mononuclear metal complex. The Mo^VI^ complex adopts a slightly distorted tetra­hedral geometry. The Mo1 N1 triple bond length is 1.633 (6) Å, which is shorter than the Mo N triple bond length in the B(C_6_F_5_)_3_ complex [1.696 (3) Å; Finke & Moore, 2010 ▸), but is very close to that in the pyridine complex [1.640 (3) Å; Chiu *et al.*, 1998 ▸). This is reasonable because the nitrido group is the terminal group in both the tile complex and the pyridine complex, whereas the nitrido group also bonds to the boron atom in the B(C_6_F_5_)_3_ complex. The Mo1—N2 bond length [1.934 (7) Å] is longer than that in the B(C_6_F_5_)_3_ complex [1.914 (3) Å], but is shorter than that in the pyridine complex [1.973 (3) Å]. The Mo1—O1 bond length [1.886 (3) Å] is longer than those in the B(C_6_F_5_)_3_ complex [1.838 (3), 1.839 (3) Å], but is shorter than those in the pyridine complex [1.921 (3), 1.924 (2) Å]. It is also reasonable that the Mo1—N2 and Mo1—O1 bonds are strengthened in the B(C_6_F_5_)_3_ complex, but weakened in the pyridine complex. In the B(C_6_F_5_)_3_ complex, the Mo1 N1 bond is weakened due to the formation of a N1→B bond. Therefore, the rest of the bonds to the central Mo atom are strengthened. In the pyridine complex, however, the central Mo atom is five-coordinated with an extra bond between Mo and the nitro­gen atom of pyridine. Since the Mo1 N1 triple bond is retained, the Mo1—N2 and Mo1—O1 bonds are weakened. Our assumption is that the central Mo atom has the same valence (+VI) in all three compounds.

## Supra­molecular features   

**Figure 2:**
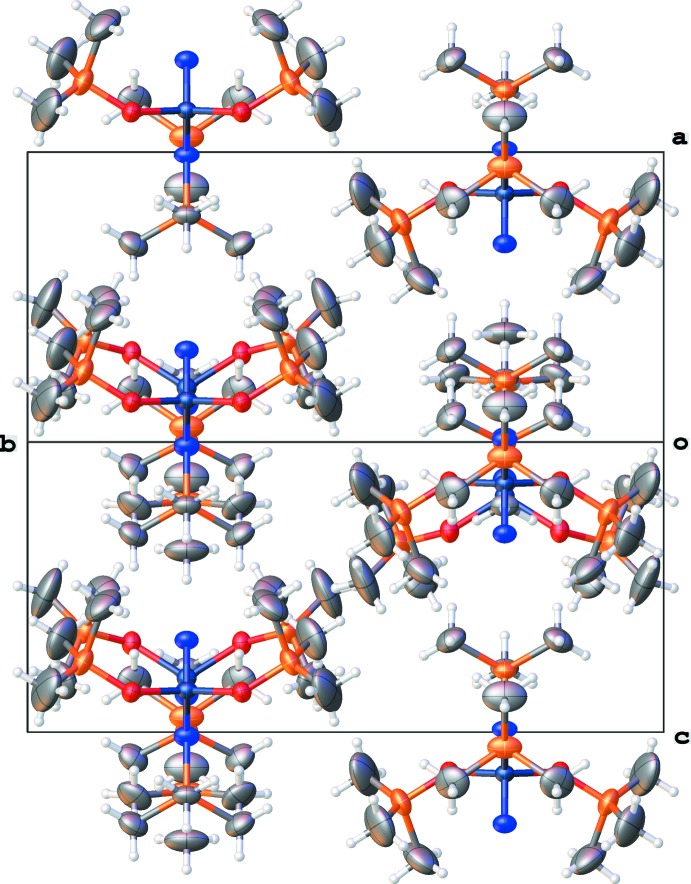
The packing of the title compound viewed along [101]. Displacement ellipsoids are drawn at the 30% probability level.

No hydrogen bonding is observed in the crystal structure. The packing of the molecules is depicted in Fig. 2. ▸

## Database survey   

The crystal structures of two similar compounds, *i.e*. the pyridine and tris­(penta­fluoro­phen­yl)borane complexes of the title compound, have been reported by Chiu *et al.* (1998 ▸) and Finke & Moore (2010 ▸), respectively. The crystal structures of other related nitridomolybdenum complexes include nitridomolybdenum complexes with alk­oxy (Chan *et al.*, 1986 ▸; Gdula *et al.*, 2005 ▸; Finke & Moore, 2010 ▸; Wiedner *et al.*, 2011 ▸), sil­yloxy (Kim & DeKock, 1989 ▸; Chiu *et al.*, 1996 ▸; Bindl *et al.*, 2009 ▸; Heppekausen *et al.*, 2010 ▸; Heppekausen *et al.*, 2012 ▸), ar­yloxy (Zeller *et al.*, 2005 ▸; Wiedner *et al.*, 2011 ▸), amido (Gebeyehu *et al.*, 1991 ▸; Kim *et al.*, 1994 ▸; Laplaza *et al.*, 1996 ▸; Tsai *et al.*, 1999 ▸; Sceats *et al.*, 2004 ▸; Figueroa *et al.*, 2006 ▸; Curley *et al.*, 2008 ▸; Yandulov *et al.*, 2003 ▸; Wampler & Schrock, 2007 ▸; Reithofer *et al.*, 2010 ▸; DiFranco *et al.*, 2013 ▸) and other ligands (Caulton *et al.*, 1995 ▸; Peters *et al.*, 1996 ▸; Agapie *et al.*, 2000 ▸; Chisholm *et al.*, 2002 ▸; Sarkar *et al.*, 2008 ▸).

## Synthesis and crystallization   

The title compound was synthesized according to a literature method (Bindl *et al.*, 2009 ▸). A flask was charged with Na_2_MoO_4_ (40 mmol, 8.24 g), Me_3_SiCl (160 mmol, 20.4 mL) and freshly distilled 1,2-di­meth­oxy­ethane (280 mL). The mixture was vigorously stirred under reflux for 16 h under N_2_. After cooling to room temperature, the white suspension in the flask was place into a glove box filled with Ar. The solvent was evaporated and the light-blue residue suspended in freshly distilled hexane (280 ml). Solid LiN(SiMe_3_)_2_ (80 mmol, 13.4 g) was added in three portions over 1 h to the suspension. The brownish green mixture was stirred at room temperature for a further 4 h. For work-up, the suspension was filtered through a pad of Celite under Ar, the brown filtrate was concentrated and the residue distilled under high vacuum to give the title compound as a light-brown oil (3.4 g, 19% yield based on Na_2_MoO_4_). This oily product was left at 288 K for several days to give colorless crystals suitable for single-crystal X-ray diffraction.

The crystals were first examined under a microscope. In order to avoid melting and reacting with air and moisture, crystals had to be submerged in several drops of inert oil cooled by ice. Then the selected crystal was quickly (less than 2 seconds) transferred to the cold nitro­gen flow of the diffractometer. Initially, data collection was completed at 173 K. However, the final reduced data were not satisfactory. The unit-cell parameters were similar to those in Table 1 ▸, but *R*_merge_ was around 0.1. We suspected that there might be some kind of phase transition at 173 K, but did not perform any further investigations. By setting the temperature to 248 K, we found that the single crystal was stable, and the diffraction spots/patterns appeared acceptable. Therefore, data collection was completed at 248 K.

## Refinement   

Crystal data, data collection and structure refinement details are summarized in Table 1 ▸. The title complex resides on a crystallographic mirror plane. Therefore, only half of the complex is unique. Atoms Mo1, N1, N2, Si2, Si3, C4, and C6 lie on the mirror plane exactly. The Si(CH_3_)_3_ groups are highly disordered in the structure. Therefore, it is probably inappropriate to split the Si(CH_3_)_3_ group into two parts. Instead, the Si(CH_3_)_3_ groups are modeled in an ordered way, as if they are not disordered. In consequence, the Si—C bond lengths differ quite largely, and the ADPs of the methyl carbons are very eccentric. Therefore, several restraints were used including ‘SADI 0.01 Si1 C1 Si1 C2 Si1 C3 Si2 C4 Si2 C5 Si3 C6 Si3 C7’ (similar Si—C bond length) and ‘ISOR 0.01 0.02 C1 C2 C3 C6 C7’ (isotropic ADPs approximately). The C-bound H atoms were placed in calculated positions and treated as riding atoms: C—H = 0.97 Å with *U*_iso_(H) = 1.5*U*_eq_(C).

## Supplementary Material

Crystal structure: contains datablock(s) I, global. DOI: 10.1107/S2056989015021192/xu5878sup1.cif

Structure factors: contains datablock(s) I. DOI: 10.1107/S2056989015021192/xu5878Isup2.hkl

Supporting information file. DOI: 10.1107/S2056989015021192/xu5878Isup3.cdx

CCDC reference: 1432821

Additional supporting information:  crystallographic information; 3D view; checkCIF report

## Figures and Tables

**Table 1:** Experimental details

Crystal data	
Chemical formula	[Mo(C_6_H_18_NSi_2_)(C_3_H_9_OSi)_2_N]
*M*_r_	448.73
Crystal system, space group	Orthorhombic, *P* *n* *m* *a*
Temperature (K)	248
*a*, *b*, *c* (Å)	10.685 (2), 18.236 (4), 13.228 (3)
*V* (Å^3^)	2577.5 (9)
*Z*	4
Radiation type	Mo *K*α
μ (mm^−1^)	0.70
Crystal size (mm)	0.50 × 0.45 × 0.42

Data collection	
Diffractometer	Rigaku Saturn724+ CCD
Absorption correction	Multi-scan (*CrystalClear*; Rigaku, 2008 ▸)
*T*_min_, *T*_max_	0.417, 1.000
No. of measured, independent and observed [*I* > 2σ(*I*)] reflections	9635, 2995, 2801
*R*_int_	0.046
(sin θ/λ)_max_ (Å^−1^)	0.648

Refinement	
*R*[*F* ^2^ > 2σ(*F* ^2^)], *wR*(*F* ^2^), *S*	0.068, 0.195, 1.19
No. of reflections	2995
No. of parameters	106
No. of restraints	51
H-atom treatment	H-atom parameters constrained
Δρ_max_, Δρ_min_ (e Å^−3^)	0.72, −0.73

Computer programs: *CrystalClear* (Rigaku, 2008 ▸), *SHELXS2013* (Sheldrick, 2008 ▸), *SHELXL2014* (Sheldrick, 2015 ▸), *OLEX2* (Dolomanov *et al.*, 2009 ▸) and *CIFTAB* (Sheldrick, 2008 ▸).

## supplementary crystallographic information

### Crystal data

**Table:**

[Mo(C_6_H_18_NSi_2_)(C_3_H_9_OSi)_2_N]	*D*_x_ = 1.156 Mg m^−^^3^
*M**_r_* = 448.73	Mo *K*α radiation, λ = 0.71073 Å
Orthorhombic, *P**n**m**a*	Cell parameters from 8012 reflections
*a* = 10.685 (2) Å	θ = 2.5–27.4°
*b* = 18.236 (4) Å	µ = 0.70 mm^−^^1^
*c* = 13.228 (3) Å	*T* = 248 K
*V* = 2577.5 (9) Å^3^	Block, colorless
*Z* = 4	0.50 × 0.45 × 0.42 mm
*F*(000) = 944	

### Data collection

**Table:**

Rigaku Saturn724+ CCD diffractometer	2995 independent reflections
Radiation source: Sealed Tube	2801 reflections with *I* > 2σ(*I*)
Detector resolution: 28.5714 pixels mm^-1^	*R*_int_ = 0.046
ω scans at fixed χ = 45°	θ_max_ = 27.4°, θ_min_ = 3.3°
Absorption correction: multi-scan (*CrystalClear*; Rigaku, 2008)	*h* = −9→13
*T*_min_ = 0.417, *T*_max_ = 1.000	*k* = −23→12
9635 measured reflections	*l* = −17→17

### Refinement

**Table:**

Refinement on *F*^2^	51 restraints
Least-squares matrix: full	Hydrogen site location: inferred from neighbouring sites
*R*[*F*^2^ > 2σ(*F*^2^)] = 0.068	H-atom parameters constrained
*wR*(*F*^2^) = 0.195	*w* = 1/[σ^2^(*F*_o_^2^) + (0.0923*P*)^2^ + 2.659*P*] where *P* = (*F*_o_^2^ + 2*F*_c_^2^)/3
*S* = 1.19	(Δ/σ)_max_ = 0.003
2995 reflections	Δρ_max_ = 0.72 e Å^−^^3^
106 parameters	Δρ_min_ = −0.73 e Å^−^^3^

### Special details

**Table:**

Geometry. All e.s.d.'s (except the e.s.d. in the dihedral angle between two l.s. planes) are estimated using the full covariance matrix. The cell e.s.d.'s are taken into account individually in the estimation of e.s.d.'s in distances, angles and torsion angles; correlations between e.s.d.'s in cell parameters are only used when they are defined by crystal symmetry. An approximate (isotropic) treatment of cell e.s.d.'s is used for estimating e.s.d.'s involving l.s. planes.

### Fractional atomic coordinates and isotropic or equivalent isotropic displacement parameters (Å^2^)

**Table:**

	*x*	*y*	*z*	*U*_iso_*/*U*_eq_	
Mo1	0.66543 (5)	0.7500	0.47814 (4)	0.0529 (2)	
Si1	0.7956 (2)	0.58706 (10)	0.44615 (17)	0.0902 (6)	
Si2	0.39517 (18)	0.7500	0.39202 (15)	0.0622 (5)	
Si3	0.4241 (2)	0.7500	0.62778 (19)	0.1028 (10)	
O1	0.7220 (3)	0.66325 (17)	0.4154 (3)	0.0663 (8)	
N1	0.7248 (6)	0.7500	0.5919 (5)	0.0750 (17)	
N2	0.4871 (6)	0.7500	0.5029 (4)	0.0657 (15)	
C1	0.9544 (8)	0.6185 (7)	0.4818 (10)	0.196 (6)	
H1A	1.0049	0.5767	0.5013	0.294*	
H1B	0.9929	0.6430	0.4246	0.294*	
H1C	0.9485	0.6524	0.5381	0.294*	
C2	0.7505 (14)	0.5537 (6)	0.5716 (7)	0.203 (6)	
H2A	0.7959	0.5089	0.5866	0.305*	
H2B	0.7705	0.5906	0.6219	0.305*	
H2C	0.6613	0.5439	0.5728	0.305*	
C3	0.8115 (14)	0.5296 (6)	0.3335 (8)	0.255 (8)	
H3A	0.8547	0.4846	0.3510	0.383*	
H3B	0.7291	0.5180	0.3072	0.383*	
H3C	0.8590	0.5558	0.2825	0.383*	
C4	0.4993 (7)	0.7500	0.2804 (5)	0.080 (2)	
H4A	0.4491	0.7500	0.2193	0.119*	
H4B	0.5517	0.7066	0.2817	0.119*	
C5	0.2968 (7)	0.6661 (4)	0.3858 (7)	0.117 (3)	
H5A	0.2470	0.6670	0.3245	0.175*	
H5B	0.3502	0.6231	0.3855	0.175*	
H5C	0.2419	0.6644	0.4441	0.175*	
C6	0.2517 (7)	0.7500	0.6258 (11)	0.170 (6)	
H6A	0.2202	0.7500	0.6945	0.255*	
H6B	0.2221	0.7066	0.5908	0.255*	
C7	0.4771 (8)	0.6677 (4)	0.6971 (6)	0.143 (3)	
H7A	0.4424	0.6682	0.7648	0.214*	
H7B	0.4490	0.6240	0.6619	0.214*	
H7C	0.5678	0.6676	0.7009	0.214*	

### Atomic displacement parameters (Å^2^)

**Table:**

	*U*^11^	*U*^22^	*U*^33^	*U*^12^	*U*^13^	*U*^23^
Mo1	0.0528 (4)	0.0577 (4)	0.0483 (3)	0.000	−0.0068 (2)	0.000
Si1	0.1046 (13)	0.0658 (9)	0.1000 (13)	0.0228 (9)	0.0059 (11)	0.0208 (9)
Si2	0.0523 (10)	0.0781 (12)	0.0563 (10)	0.000	−0.0071 (8)	0.000
Si3	0.0779 (16)	0.177 (3)	0.0534 (12)	0.000	0.0106 (11)	0.000
O1	0.073 (2)	0.0543 (17)	0.072 (2)	0.0035 (16)	−0.0098 (17)	0.0003 (15)
N1	0.078 (4)	0.085 (4)	0.062 (3)	0.000	−0.020 (3)	0.000
N2	0.051 (3)	0.097 (4)	0.049 (3)	0.000	−0.007 (2)	0.000
C1	0.134 (8)	0.189 (11)	0.265 (13)	0.050 (9)	−0.032 (8)	0.061 (9)
C2	0.261 (13)	0.140 (8)	0.209 (11)	0.017 (9)	0.030 (11)	0.097 (8)
C3	0.394 (17)	0.138 (8)	0.233 (14)	0.132 (10)	−0.047 (11)	−0.063 (9)
C4	0.069 (4)	0.119 (6)	0.050 (3)	0.000	−0.010 (3)	0.000
C5	0.100 (5)	0.128 (6)	0.121 (6)	−0.041 (5)	−0.012 (5)	−0.001 (5)
C6	0.116 (9)	0.294 (15)	0.101 (8)	0.000	0.035 (7)	0.000
C7	0.176 (8)	0.173 (8)	0.080 (4)	−0.033 (7)	0.018 (5)	0.043 (5)

### Geometric parameters (Å, º)

**Table:**

Mo1—N1	1.633 (6)	C1—H1C	0.9700
Mo1—N2	1.934 (7)	C2—H2A	0.9700
Mo1—O1^i^	1.886 (3)	C2—H2B	0.9700
Mo1—O1	1.886 (3)	C2—H2C	0.9700
Si1—O1	1.648 (4)	C3—H3A	0.9700
Si1—C3	1.830 (8)	C3—H3B	0.9700
Si1—C2	1.832 (7)	C3—H3C	0.9700
Si1—C1	1.852 (8)	C4—H4A	0.9700
Si2—N2	1.766 (6)	C4—H4B	0.9700
Si2—C4	1.849 (6)	C5—H5A	0.9700
Si2—C5	1.859 (6)	C5—H5B	0.9700
Si2—C5^i^	1.859 (6)	C5—H5C	0.9700
Si3—N2	1.783 (7)	C6—H6A	0.9700
Si3—C6	1.842 (8)	C6—H6B	0.9700
Si3—C7^i^	1.848 (6)	C7—H7A	0.9700
Si3—C7	1.848 (6)	C7—H7B	0.9700
C1—H1A	0.9700	C7—H7C	0.9700
C1—H1B	0.9700		
			
N1—Mo1—O1^i^	106.33 (16)	H1A—C1—H1C	109.5
N1—Mo1—O1	106.34 (16)	H1B—C1—H1C	109.5
O1^i^—Mo1—O1	114.0 (2)	Si1—C2—H2A	109.5
N1—Mo1—N2	103.1 (3)	Si1—C2—H2B	109.5
O1^i^—Mo1—N2	112.98 (13)	H2A—C2—H2B	109.5
O1—Mo1—N2	112.98 (13)	Si1—C2—H2C	109.5
O1—Si1—C3	109.0 (4)	H2A—C2—H2C	109.5
O1—Si1—C2	112.2 (4)	H2B—C2—H2C	109.5
C3—Si1—C2	124.9 (6)	Si1—C3—H3A	109.5
O1—Si1—C1	103.8 (4)	Si1—C3—H3B	109.5
C3—Si1—C1	107.4 (7)	H3A—C3—H3B	109.5
C2—Si1—C1	96.5 (6)	Si1—C3—H3C	109.5
N2—Si2—C4	109.2 (3)	H3A—C3—H3C	109.5
N2—Si2—C5	110.6 (3)	H3B—C3—H3C	109.5
C4—Si2—C5	107.8 (3)	Si2—C4—H4A	109.5
N2—Si2—C5^i^	110.6 (3)	Si2—C4—H4B	109.5
C4—Si2—C5^i^	107.8 (3)	H4A—C4—H4B	109.5
C5—Si2—C5^i^	110.9 (5)	Si2—C5—H5A	109.5
N2—Si3—C6	111.3 (5)	Si2—C5—H5B	109.5
N2—Si3—C7^i^	110.1 (3)	H5A—C5—H5B	109.5
C6—Si3—C7^i^	108.3 (4)	Si2—C5—H5C	109.5
N2—Si3—C7	110.1 (3)	H5A—C5—H5C	109.5
C6—Si3—C7	108.3 (4)	H5B—C5—H5C	109.5
C7^i^—Si3—C7	108.6 (6)	Si3—C6—H6A	109.5
Si1—O1—Mo1	138.7 (2)	Si3—C6—H6B	109.5
Si2—N2—Si3	124.0 (4)	H6A—C6—H6B	109.5
Si2—N2—Mo1	114.0 (3)	Si3—C7—H7A	109.5
Si3—N2—Mo1	121.9 (3)	Si3—C7—H7B	109.5
Si1—C1—H1A	109.5	H7A—C7—H7B	109.5
Si1—C1—H1B	109.5	Si3—C7—H7C	109.5
H1A—C1—H1B	109.5	H7A—C7—H7C	109.5
Si1—C1—H1C	109.5	H7B—C7—H7C	109.5
			
C3—Si1—O1—Mo1	179.5 (6)	C4—Si2—N2—Mo1	0.000 (1)
C2—Si1—O1—Mo1	36.8 (7)	C5—Si2—N2—Mo1	118.4 (3)
C1—Si1—O1—Mo1	−66.3 (6)	C5^i^—Si2—N2—Mo1	−118.4 (3)
N1—Mo1—O1—Si1	9.5 (4)	C6—Si3—N2—Si2	0.000 (1)
O1^i^—Mo1—O1—Si1	126.4 (3)	C7^i^—Si3—N2—Si2	−120.1 (3)
N2—Mo1—O1—Si1	−102.8 (4)	C7—Si3—N2—Si2	120.1 (3)
C4—Si2—N2—Si3	180.000 (1)	C6—Si3—N2—Mo1	180.000 (1)
C5—Si2—N2—Si3	−61.6 (3)	C7^i^—Si3—N2—Mo1	59.9 (3)
C5^i^—Si2—N2—Si3	61.6 (3)	C7—Si3—N2—Mo1	−59.9 (3)

Symmetry code: (i) *x*, −*y*+3/2, *z*.
